# Editorial: Immune Landscape of Kidney Pathology

**DOI:** 10.3389/fphys.2021.827537

**Published:** 2022-01-25

**Authors:** Patrick Ming-Kuen Tang, Haiyong Chen, Ying Tang, David J. Nikolic-Paterson, Hui Yao Lan

**Affiliations:** ^1^Department of Anatomical and Cellular Pathology, State Key Laboratory of Translational Oncology, Prince of Wales Hospital, The Chinese University of Hong Kong, Hong Kong, Hong Kong SAR, China; ^2^School of Chinese Medicine, Li Ka Shing Faculty of Medicine, The University of Hong Kong, Hong Kong, China; ^3^Department of Nephrology, The Third Affiliated Hospital, Southern Medical University, Guangzhou, China; ^4^Department of Nephrology, Monash University, Clayton, VIC, Australia; ^5^Department of Medicine, Monash Medical Centre, Clayton, VIC, Australia; ^6^Department of Medicine and Therapeutics, Li Ka Shing Institute of Health Sciences, Lui Che Woo Institute of Innovative Medicine, The Chinese University of Hong Kong, Hong Kong, Hong Kong SAR, China; ^7^Guangdong-Hong Kong Joint Laboratory on Immunological and Genetic Kidney Diseases, The Chinese University of Hong Kong, Hong Kong, Hong Kong SAR, China

**Keywords:** inflammation, fibrosis, immunity, therapy, lncRNA, macrophage-myofibroblast transition, COVID-19

## Introduction

Kidney disease is an emerging cause of morbidity and mortality. More than 6 million patients worldwide receive renal replacement therapy. The global prevalence of chronic kidney disease (CKD) is between 11.7 and 15.1% of the adult population. Nevertheless, we still lack effective treatments to stop the progression of CKD, which makes it an urgent area with unmet clinical need. CKD is defined as abnormal kidney structure and/or function caused by primary and secondary glomerular diseases (including diabetes, hypertension, autoimmune diseases, etc.). Renal fibrosis is a common feature of CKD and is widely regarded as the main driver of the progression to end-stage renal disease. However, the underlying mechanisms of the renal fibrotic response are complex and still poorly understood. Emerging research shows that unresolved inflammation may be a necessary condition to promote the transition from acute kidney injury to chronic renal fibrosis.

Various white blood cell populations are recruited into injured kidneys and play important roles in pathogen clearance and tissue repair. However, if this inflammatory response does not subside, it will instead promote progressive fibrosis of the damaged kidney. Interestingly, a large number of studies have shown that infiltrating leukocytes, including macrophages, dendritic cells, natural killer cells, and T and B cells, actively promote the transition from renal inflammation to fibrosis (Tang et al., 2020a). In addition, changes in the microenvironment in different kidney compartments also play a key role in the immune response and disease pathogenesis. A better understanding of the immune process in the development of CKD may reveal direct and indirect immunomodulation methods as new therapeutic strategies to prevent the progression of different forms of kidney disease.

Therefore, we initiated this research project co-sponsored by Frontiers in Physiology and Frontiers in Medicine, aiming to bring together research from multiple disciplines, with special attention to immunology, renal physiology and pathology. We invited researchers to share their latest insights into how host immunity and its effectors reshape the kidney microenvironment to achieve the physiological and/or pathogenic effects of diseased kidneys.

We are very pleased that this Research Topic has been welcomed by basic researchers and clinical scientists from all over the world. A total of 22 high-quality papers have been published, including nine original studies, six reviews, four mini-reviews, one case report and a brief research report. These papers are written by 159 authors from around the world, providing cross-sectional and multi-disciplinary approaches in the latest kidney disease research. Broadly speaking, these papers focus on five core topics: (i) immunodynamics; (ii) pathogenic mechanisms; (iii) advanced research technology; (iv) therapeutic development; and (v) social impact on patients with kidney disease. The following is a brief overview of each study.

### Immunodynamics

The kidney is one of the main organs for detoxification in our body. Its failure is an important cause of patient death. In addition, kidney disease is a major contributor to patient death in a wide range of diseases such as diabetes, cancer, bacterial and viral infections (including COVID-19; Tang et al., 2021a; Wang et al., 2021); leading to more than 6 million deaths worldwide each year. Thus, developing a better understanding, and treatment of, kidney disease is critical. Renal fibrosis is a key pathological mechanism in the loss of normal structure and function of the kidney, resulting in progressive kidney damage. Encouragingly, scientists have begun to realize that the over-activation of the immune system is an essential component in this process, and this feature is summarized by Tang et al. in this Research Topic.

**Figure 1:**
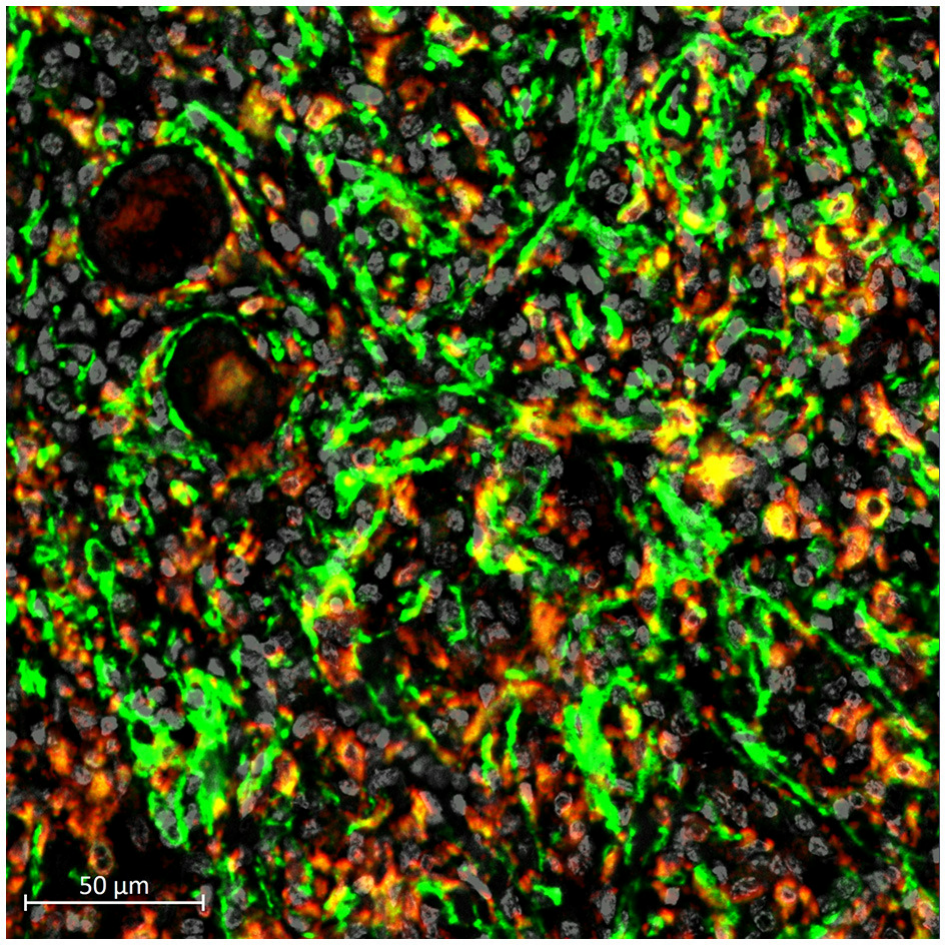
Occurrence of MMT (yellow) in a human kidney with chronic allograft dysfunction, indicating by the presence of macrophage (CD68, red) expressing myofibroblast marker (alpha-SMA, green).

Macrophages are a type of immune cell that maintains the health of our kidneys (Tang et al., 2019). They are responsible for detecting, engulfing, and destroying pathogens and unhealthy cells as discussed by Cantero-Navarro et al. Paradoxically, new research finds that macrophages can also accelerate kidney failure as highlighted by a systematic review from Wang et al. A better understanding of the underlying mechanisms can isolate the adverse effects of macrophages from their protective effects. For example, a new phenomenon “macrophage-myofibroblast transition (MMT)” has been identified as a pathway promoting the tissue scarring (Figure 1), and dissecting this MMT pathway may identify novel druggable therapeutic targets for kidney fibrosis (Tang et al., 2018a, 2020b).

Changes in the immune landscape are essential components in both disease pathogenesis and tissue repair in states of inflammation, but much remains to be done to fully describe such changes in kidney diseases. Vonbrunn et al. investigated the potential significance of glomerular immune reactivity for allograft survival by analyzing the immune profile of time zero kidney specimens and how this impact clinical outcomes. Albino et al. elucidated how innate immunity contributes to the transition of acute kidney injury to renal fibrosis in a gentamicin-induced renal inflammation model. Furthermore, Rodriguez-Carrio et al. found changes in several novel T cell and monocyte subsets during the progression of chronic kidney disease which were significantly associated with vascular outcomes.

### Pathogenic Mechanisms

Unresolved renal inflammation can drive the progression of renal fibrosis, leading to end-stage renal disease. Understanding the mechanisms underlying this unrelenting renal fibrosis is critical for the development of new therapies to halt disease progression. Shao et al. described how a variety of key signaling molecules (e.g., TGF-β1, NF-κB, MAPK, NLRPs, etc.) and epigenetic changes (e.g., DNA methylation, histone modification, and expression of non-coding RNA) contributes to renal inflammation in the pathogenesis of diabetic kidney disease (Tang et al., 2018b; Chung et al., 2021).

Acute kidney injury (AKI) can lead to progressive kidney disease. A number of novel long non-coding RNAs have been identified that contribute to both the development of AKI and in the progression of CKD (Sun et al., 2018; Zhang et al., 2019). Yang et al. revealed that JNK signaling causes aristolochic acid-induced renal tubular cell damage. Zhang et al. identified that lncRNA LRNA9884 enhances the release of inflammatory cytokines through the NF-κB pathway after cisplatin-induced AKI and promotes renal inflammation by binding to the *Ccl2* promotor in the *db/db* mouse model of type-2 diabetes (Zhang et al., 2019).

In C3 glomerulonephritis, the D288G mutation in the gene encoding complement factor I was shown to contribute to C3 deposition in mesangial cells (Song et al.). In renal vasculitis, Tan et al. found that systemic glomerulosclerosis and segmental sclerosis are prognostic and therapeutic markers of IgA vasculitis with nephritis. In addition, new signaling pathways have been identified which act in a cell-type and disease-specific manner in experimental models and kidney patients to promote the progression of kidney disease (Tang et al., 2021a). This basic research work provides examples of how understanding the pathogenesis of kidney disease at the molecular level has the potential to develop precision medicine for kidney disease.

### Advanced Research Technology

Understanding the highly dynamic nature of the renal microenvironment during disease development and progression is a major challenge. Encouragingly, there are a number of hallmark analytic technologies have been developed in the last decade, which significantly facilitate and accelerate research into kidney disease in a multidisciplinary manner (Park et al., 2018).

Clusters of regularly spaced short palindromic repeats (CRISPR)—CRISPR-associated protein 9 (Cas9) is an RNA-guided DNA nuclease that has been used to develop simple and efficient techniques to precisely engineer the genome. The CRIPSR-Cas9 system has been widely used to simultaneously delete multiple genes, create conditional alleles, and generate reporter proteins *in vitro* as well as *in vivo* (Higashijima et al., 2017). By using the latest genome editing platform CRISPR/Cas9, Song et al. effectively characterize the mutations of complement factors in a mouse model with C3 glomerulopathy.

Single-cell RNA-sequencing is a breakthrough in biological research for elucidating changes at the single cell level and understanding cell-cell interactions in the complex microenvironment of both physiological conditions and disease development. It is particularly suited to dissect the immunodynamics of kidney disease. In this Research Topic, Zeng et al. systematically summarized the development and application of single-cell RNA-sequencing in kidney immunology. Interestingly, recent work has revealed an unexpected role of macrophage-myofibroblast transition, first identified in kidney fibrosis, in promoting tumor development through tumor-associated macrophage transitioning into cancer-associated fibroblasts in non-small-cell lung carcinoma (Tang et al., 2021b), suggesting an important contribution of tissue fibrotic pathways in cancer. Therefore, we also opened a new platform in Frontiers for sharing the new insights into fibrotic signaling in cancer (https://www.frontiersin.org/research-topics/22920/new-insights-into-fibrotic-signaling-in-cancer).

### Therapeutic Development

Several papers in this Research Topic describe therapeutic strategies to inhibit inflammation and immune cell function in kidney disease. Protein kinases are a large family of enzymes that regulate many intracellular signaling processes. For example, spleen tyrosine kinase (SYK) is required for signaling *via* cell surface receptors involved in inflammation, including immunoglobulin receptors. SYK signaling occurs in inflammatory forms of human kidney diseases, and genetic or drug-based SYK inhibition is protective in animal models of crescentic glomerulonephritis and antibody-mediated kidney allograft rejection (Ryan et al., 2016; Ramessur Chandran et al., 2017). Yiu et al. show that SYK is activated in tubular epithelial cells in patients with IgA nephropathy, and that polymeric IgA from patients with IgA nephropathy (but not from healthy controls) activates an inflammatory response in cultured tubular epithelial cells *via* SYK—identifying a SYK-dependent mechanism of tubulointerstitial inflammation.

The JUN amino-terminal kinase (JNK) is a widely expressed enzyme that is highly sensitive to activation by oxidative stress and DNA damage. Activated JNK can phosphorylate protein targets to promote cell necrosis, inflammation and fibrosis (Grynberg et al., 2017). Yang et al. show that the nephrotoxin aristolochic acid—the cause of Chinese herb nephropathy and Balkan nephropathy—induces DNA damage and prominent JNK activation in tubular epithelial cells in mice. Treatment with a JNK inhibitor provided significant protection against tubular necrosis, macrophage infiltration, inflammation and acute renal failure in response to acute high dose AA administration. This further supports therapeutic targeting of JNK to prevent acute kidney injury.

While small molecule drugs are the backbone of current therapies for kidney disease, stem cells and extracellular vesicles are being developed as new potential treatments. Human amniotic epithelial cells (hAEC) are an attractive therapy due to their immunosuppressive capacity, their lack of immunogenicity and their ready availability—being isolated from the human placenta after birth (Al Mushafi et al.). Their immunosuppressive capacity is attributed to secretion of IL-10, TGF-β1, PGE2 and exosomes, while hAEC also act to increase numbers of Tregs and Th2 T cells. Treatment with hAEC suppressed autoantibody production and reduced levels of IL-17 and IFN-g in a mouse model of lupus nephritis (Tan et al., 2018).

Exosomes are a class of small extracellular vesicles excreted by most cell types (Shen et al.). Exosomes carry a cargo of RNA and proteins which, upon uptake into recipient cells, can modulate cell function. Treatment with specific exosome populations can suppress immune-mediated acute and chronic kidney disease models (Eirin and Lerman, 2021). In addition, since the contents of exosomes reflects the cell type of origin, exosomes are being investigated as novel biomarkers in human kidney disease. Furthermore, by manipulating the receptors in the exosome membrane, it is possible to target exosomes to specific cell types—opening up the potential for cell-directed delivery of exosomes carrying biological molecules or drugs to modify disease progression (Shen et al.).

### Social Impact

Since the first case in 2019, the COVID-19 pandemic remains an unresolved global issue (Worobey, 2021), among which acute kidney injury is one of the complications of patients infected with the virus (Huang et al., 2020). In fact, social issues not only cause physical harm to humans, but may also affect patients with chronic diseases psychologically.

In this Research Topic, Chan et al. studied the impact of the COVID-19 pandemic on the mental health of patients, revealing a significant impact on the quality of life of patients with chronic kidney diseases receiving dialysis. In addition, Nie et al. conducted a multicentre retrospective cohort study which identified the necessity of kidney biopsy collection for an accurate diagnosis of patients with monoclonal gammopathy. Another retrospective study conducted by Hakroush et al. emphasized the need for histopathological findings in order to make better treatment decisions for critically ill patients who have already exhibited worsening renal function.

## Summary

In summary, these papers outline research on the importance of the immune landscape in kidney disease, showing the clinical significance and translational potential of the Research Topic, and providing insights into many exciting research avenues. Our understanding of kidney disease is the immune landscape in kidney pathogenesis continues to grow.

## Author Contributions

PT and DN-P have made a substantial, direct, and intellectual contribution to the work. HC, YT, and HL edited and approved it for publication.

## Funding

This collaborative work was supported by Research Grants Council of Hong Kong (14106518, 14111019, and 14111720) and the Chinese University of Hong Kong's Faculty Innovation Award (4620528).

## Conflict of Interest

The authors declare that the research was conducted in the absence of any commercial or financial relationships that could be construed as a potential conflict of interest.

## Publisher's Note

All claims expressed in this article are solely those of the authors and do not necessarily represent those of their affiliated organizations, or those of the publisher, the editors and the reviewers. Any product that may be evaluated in this article, or claim that may be made by its manufacturer, is not guaranteed or endorsed by the publisher.
